# Healthy Aging Promotion through Neuroscientific Information-Based Strategies

**DOI:** 10.3390/ijerph121012158

**Published:** 2015-09-28

**Authors:** Sofia Seinfeld, Maria V. Sanchez-Vives

**Affiliations:** 1 Institut d’Investigacions Biomèdiques August Pi i Sunyer (IDIBAPS), 08036 Barcelona, Spain; E-Mail: msanche3@clinic.ub.es; 2 Event Lab, Faculty of Psychology, University of Barcelona, 08035 Barcelona, Spain; 3 Institució Catalana de Recerca i Estudis Avançats (ICREA), 08010 Barcelona, Spain; 4 Department of Basic Psychology, University of Barcelona, 08035 Barcelona, Spain

**Keywords:** brain, health, lifestyle, aging, brain plasticity, information, healthy aging, brain health, cognition, active aging

## Abstract

To ensure the well-being of a rapidly growing elderly population, it is fundamental to find strategies to foster healthy brain aging. With this intention, we designed a program of scientific-based lectures aimed at dissemination by established neuroscientists about brain function, brain plasticity and how lifestyle influences the brain. We also carried out a pilot study on the impact of the lectures on attendees. The objective was to provide information to elderly people in order to encourage them to identify unhealthy and healthy daily habits, and more importantly, to promote behavioral changes towards healthy brain aging. Here we report on our experience. In order to determine the impact of the lectures in the daily routine of the attendees, we asked them to fill out questionnaires. Preliminary results indicate that neuroscientific information-based strategies can be a useful method to have a positive impact on the lives of elderly, increase their awareness on how to improve brain function and promote positive lifestyle modifications. Furthermore, based on self-reported data, we also found that through this strategy it is possible to promote behavioral changes related to nutrition, sleep, and realization of physical and cognitively stimulating activities. Finally, based on the results obtained, the importance of promoting self-efficacy and the empowerment of the older populations is highlighted.

## 1. Introduction

The United Nations has estimated that the number of older adults worldwide will more than triple by the year 2050, when it will surpass the two billion mark [1]. To ensure the wellbeing of a rapidly growing elderly population, it is fundamental to find strategies to foster healthy lifestyle and prevent the onset of non-communicable and neurodegenerative diseases in latter stages of life.

Several studies have found a negative correlation between healthy lifestyle and mortality or the development of non-communicable diseases (for a review see [2]). In this sense, epidemiological studies have demonstrated that a healthy lifestyle plays a crucial role in the primary prevention of several diseases and is related to healthy aging. Healthy aging is defined as the process by which older adults optimize their opportunities for improving and preserving their cognitive, social and physical functioning, in order to increase their quality of life, promote their autonomy, and enhance successful life-course transitions [3]. A relevant concept for healthy aging is that of health literacy, defined as the skill which determines the degree of motivation and the ability of individuals to access, understand, and use information in ways which promote and maintain a good health [4]. It has been shown that older individuals with inadequate levels of health literacy report poorer physical and mental health functioning and greater limitations in their routine activities, even after controlling for demographics characteristics, health risk factors, and chronic conditions [5].

When designing strategies which aim to promote an improvement in different lifestyle factors and increase health literacy, it is important to consider that targeting risk factors in isolation is a less effective and efficient strategy than addressing multiple health behaviors in parallel [6]. Furthermore, when deciding the target population of these health promotion strategies, it is also important to take into account that initiating changes towards a healthy lifestyle may not only be beneficial in younger ages, but also when daily life modifications are started in middle and older ages [7,8]. Based on this evidence, it is essential to find strategies that target an elderly population, increase health literacy, and that address changes in multiple health behaviors in parallel.

Concerning lifestyle factors, which play a critical role in health, research has found a reduced all-cause mortality of 56% in non-smoking adults, 47% in physically active adults, and 26% in adults that had a healthy diet [9]. Factors like nutrition, physical activity and non-smoking are the most typical behaviors linked to health promotion [10]. However, over the last few years, it has been demonstrated that other behaviors or habits can also have a significant impact on reducing mortality. Factors such as having an adequate sleep duration, avoiding sitting for an excessive time, practicing cognitively stimulating activities and having rich social interactions might be important contributors to survival and good quality of life [11].

A series of physical and cognitive functioning declines are triggered by normal aging and have a significant impact on the lives of the elderly [12]. Several studies have shown the existence of a linear decline in executive functions, processing speed, memory, motor performance, white matter integrity and brain volume, all aligned with age [13,14,15]. However, the degree of cognitive decline and neurological changes suffered by the elderly is not homogenous. While a substantial number of older adults undergo a process of healthy brain aging evidenced by the fact that they maintain their cognitive functioning throughout the lifespan or just show modest losses, others suffer serious cognitive declines and in many cases neurological conditions as mild cognitive impairment or dementias [16].

In this regard, engaging in late-life cognitive stimulating activities (e.g., reading, writing, crossword puzzles, board or card games, group discussions or playing a musical instrument) can result in a 50% reduced risk of developing dementia and reduce the onset of accelerated memory decline [17,18]. Furthermore, it has been largely shown in the literature that having an adequate diet and practicing physical exercise in older ages not only benefits cardiovascular health and prevents several diseases, but it can also influence neuronal function and synaptic plasticity by modulating brain-derived neurotrophic factors [19,20]. Finally, researchers have also shown that a fundamental benefit of good sleep habits is promoting memory consolidation and learning [21,22]. The brain's ability to actively cope with brain damage by using pre-existing cognitive resources (or through reorganization of neuronal structures via brain plasticity) might provide evidence of how healthy lifestyle benefits brain aging (see review in [23,24]).

Community interventions might be an effective method to foster brain health, promote health literacy, and encourage changes in several health domains in parallel [25]. Based on the need of these types of interventions, which promote a healthy lifestyle in older adults, we carried out a pilot study of a conference program which we designed and named “Health and the Brain”. The program included a series of scientific-based lectures related to brain function and healthy brain aging. These lectures were delivered by leading scientists in different civic centers located in Barcelona and were open to the general public. The main goal of this pilot study was to explore if these talks, which are based on neuroscientific dissemination, can encourage elderly people to identify unhealthy and healthy daily habits, and more importantly, if they can promote behavioral changes towards healthy aging.

## 2. Experimental Section

### 2.1. Conference Program

A total of 12 lectures delivered by local senior neuroscientists in three different civic centers located in Barcelona were included in the conference program. Lectures were free and open to the general public and were advertised by the civic center’s using mailing lists, posters, and brochures given to the public. Lectures were well attended; the public was estimated by the center to be between 2/3 to full capacity, resulting in approximately 50–80 people per conference. Attendants could go to as many talks as they wanted. Table 1 summarizes the topics and lectures included in the series.

**Table 1:** Summary of topics and lectures included in the program.

Topics	Lectures
Healthy nutrition	•Food and health
	•Neuroscience in the kitchen
	•Cook for your brain
Physical activity	•Physical exercise and the brain
	•Impact of exercise on the brain and the importance of exercising to promote a healthy brain
Notions of brain plasticity and how lifestyle influences the brain	•Exercising the brain by playing a musical instrument
	•The music and the brain
	•Brain processing of language and impact of language learning on cognition
	•Healthy lifestyle and cerebrovascular health and pathologies
	•Active aging: brain plasticity in the older adult brain
Other topics	•Depression, a disease of our time
	•Sleeping and learning: effects of sleeping on the brain

### 2.2. Measures

#### 2.2.1. Demographic Information

We included a questionnaire to assess participants’ sociodemographic characteristics. Specifically, we asked respondents to report their age, education, occupation, and health status (presence of a disease and medication intake). Furthermore, we also asked respondents if they play a musical instrument, speak a second language, and the frequency in which they practice cognitively stimulating and physical activities on a Likert-type scale, which included the following values: never (1), almost never (2), sometimes (3), moderately (4), frequently (5), very frequently (6), and always (7).

#### 2.2.2. Questionnaire Assessing the Impact of the Lectures

In order to assess the impact of these lectures in older adults’ knowledge and attitudes, and also to detect possible lifestyle modification after attending the talks, we developed a questionnaire that included open and multiple-choice questions. The questionnaire had a total of 13 items answered on a 5 point Likert-scale which included the following values: not at all (1), a little bit (2), moderately (3), a lot (4), and extremely (5). Concerning attitudes, we wanted to evaluate if attendants increase their knowledge about how lifestyle modification can impact healthy brain aging and also if their knowledge regarding the learning capacity of older adults was modified after they attended the lectures. With this goal in mind we included four specific questions that asked elderly respondents to evaluate their degree of likeness of the lectures program, to assess their attitudes towards improving brain function through lifestyle modifications after the attendance to the talks, and finally, we included two questions which assessed how respondents judged the learning capacity of older adults and their ability to modify their lifestyle in order to improve their brain health after the lectures (Q1, Q2, Q4, and Q13, see Table 2).

**Table 2:** The 13 multiple choice questions included in the questionnaire in order to assess the impact of the lectures on behavioral changes towards a healthy lifestyle, the knowledge of participants concerning brain health, and their motivation to practice cognitively stimulating activities as learning a musical instrument or new language.

Questionnaire Items
Q1. Do you think that these lectures helped you in order to acquire new information that may have a positive impact on your life?
Q2. After attending the lectures, do you think that you can improve your brain function through your lifestyle?
Q3. To what extent have you changed your diet habits after attending the lectures?
Q4. To what extent have your believe concerning the learning capacity in elderly (older than 60 years) has been modified after attending the lectures?
Q5. Have you modified some habits in order to promote your cerebrovascular health after attending the lectures?
Q6. To what extent have you increased the frequency with which you practice physical activity after attending the lectures?
Q7. To what extent have you increased the frequency with which you practice cognitive stimulating activities after attending the lectures?
Q8. Do you find useful and enriching the idea of playing a musical instrument in older age?
Q9. What is your level of interest to play a musical instrument like the piano?
Q10. Do you find useful and enriching the idea of learning a new language in older age?
Q11. What is your level of interest to learn a new language?
Q12. To what extent have you modified your sleeping habits in order to improve them after attending the lectures?
Q13. To what extent you believe that older adults can carry out activities or modify their lifestyle in order to promote their brain plasticity after attending the lectures?

Furthermore, since vast amount of studies have shown that factors such as nutrition, sleep, cardiovascular health, and practicing physical and cognitively stimulating activities are linked to healthy aging (see introduction), and the conference program covered all these themes, we included five questions which asked respondents to rate the degree in which they carried out behavioral changes in order to improve these domains (Q3, Q5–7, Q12, see Table 2).

Finally, since it has been recently demonstrated that learning a musical instrument or a new language can benefit healthy brain aging and specific lectures included in the program covered these themes, we decided to include four multiple-choice questions in order to assess the degree of interest and how useful did elderly found learning to play a musical instrument or a new language in older ages (Q3, Q8–11, see Table 2).

Questionnaires were given at the end of each lecture to all attendees older than 50. Respondents were encouraged to fill in the surveys at home and to send them back to our laboratory without extra-charges in any post office once they attended the last talk of the conferences program. The questionnaires were anonymous.

### 2.3. Sample

A total of 32 subjects (25 females) answered and sent the questionnaire. The mean age of respondents was 62.94 years (S.D. = 7.19; Max = 84; Min = 52), 53% of the sample were retired, the rest were active workers, 16% suffered from a chronic diseases, and 34% took some type of medication. Furthermore, focusing on education, 47% of the respondents had secondary education, 19% had vocational training studies, and 34% had a university degree or higher. The median frequency that subjects were doing some type of cognitive stimulating activity (e.g., dancing, computer-lessons, photography, learning a new language, among others) was 6 (IQR = 3; ranging from never = 1 to always = 7) on a seven point Likert scale, and 5 (IQR = 1; ranging from never = 1 to always = 7) for physical activity. Finally, the mean number of lectures that each subject attended was of 3.69 (S.D. = 3.49; Median = 3).

## 3. Results

Remarkably, 75% (24 subjects) of the respondents intended to change some health related behavior after attending the lectures. Based on questionnaire scores, on a scale ranging from 0 = nothing to 5 = extremely, attendees believed that these lectures helped them to acquire new information that could have a positive impact in their daily life (Median = 4, IQR = 1; question 1). The extent to which attendees thought that they could improve their brain function (Median = 4; IQR = 0.5; question 2) and promote brain plasticity (Median = 4, IQR = 1; 5-point Likert scale; question 13) through lifestyle modifications after attending the talks was high based on the self-reported ratings obtained from the questionnaire. Finally, their subjective beliefs about the learning capacity of persons older than 60 years, increased after attending the lectures (Median = 4, IQR = 1; 5-point Likert scale; question 4).

Concerning the motivation and usefulness of learning a new activity, such as playing a musical instrument or learning a new language, respondents answered with medium to high ratings. Specifically, questionnaire ratings reflected that they had a high to medium level of interest in learning to play a musical instrument such as the piano (Median = 3, IQR = 2; question 9), since they considered that playing a musical instrument on older ages is an enriching and useful activity (Median = 4, IQR = 1.5; question 8). In this same line, respondents also rated with high-medium scores their interest in learning a new language (Median = 4, IQR = 1; question 11) and the level they considered this activity to be useful and enriching (Median = 4, IQR = 0; question 10).

**Figure 1:**
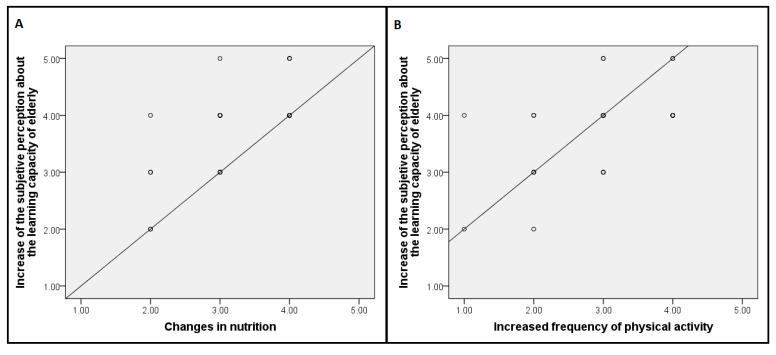
(**A**) Scatter plots showing the positive correlation between the increases of the subjective perception about the learning capacity of the elderly and the extent to which the respondents improved their nutritional habits; (**B**) Scatter plot showing the positive correlation between the increases of the subjective perception about the learning capacity of elderly and extent to which the respondents increased the frequency of practice of physical activity.

Table 3 summarizes the self-reported ratings given on questions which assessed the degree in which respondents carried out changes in order to improve their nutrition, cardiovascular health, physical activity, the practice of cognitively stimulating activities and sleep quality. As it can be seen, all of the scores tend to have medium to high ratings, which means that after attending the lectures, the respondents had already made some type of changes towards improving on these specific behaviors, although these changes were not extreme. Interestingly, we found a positive correlation between the subjective perception about the learning capacity of older adults after attending the talks (question 4) and the extent to which respondents made changes in order to improve their nutrition, r = 0.67, *p* < 0.01, and the frequency in which they practiced physical activity, r = 0.56, *p* < 0.01 (see Figure 1A,B). In order to assess if a higher number of attended lectures predicted better outcomes we run a correlation analysis. However, no significant correlations were found between the number of attended lectures and the questionnaire responses. Furthermore, a correlational analysis between the age of attendees and the questionnaire responses revealed just one significant difference. A negative correlation between the age of attendees and the degree of interest to play a musical instrument was found, r = −0.46, *p* < 0.02. This correlation indicates that participants with a more advanced age had less interest in learning a musical instrument.

**Table 3:** Summary of Medians (Md) and Interquartile Ranges (IQR) of questions (5 point Likert-type scale, ranging from 1 = nothing to 5 = extremely) assessing the level of changes done in the following behaviors after attending the lectures: eating habits, habits improving cardiovascular health, practicing physical activity, participating in cognitively stimulating activities, and sleeping habits.

Improvement of Behavior in…	Score
Eating habits	Md = 3; IQR = 1.00
Habits improving cardiovascular health	Md = 3; IQR = 1.75
Practicing physical activity	Md = 3; IQR = 1.50
Participating in cognitively stimulating activities	Md = 3; IQR = 1.75
Sleeping habits	Md = 3; IQR = 1.75

## 4. Discussion

In an aging society it is fundamental to find strategies that aim to help older adults not just to live longer, but also to maintain their quality of life and autonomy. It has been demonstrated that lifelong learning and continued education can enable elderly to keep up with scientific advances and help them to improve or maintain their quality of life by enhancing self-coping strategies in areas like nutrition, physical health, and social relationships [26]. In this exploratory study we have analyzed the impact of a series of neuroscientific information-based lectures, given by established neuroscientists, on the knowledge and brain health-related behaviors of older adults. The lectures were very well attended, mostly by the elderly, revealing the large interest that brain health raises in this segment of the population. Preliminary results indicate that most of the respondents carried out some changes in health related behavior after attending the lectures, indicating that the main goal of improving older people's lifestyle in order to prevent the onset of neurological diseases and foster brain health, was partially fulfilled. These lectures highlighted the fact that staying active physically and mentally, as well as having an adequate nutrition and practicing physical exercise, can play an important role in preserving and even boosting brain function (for review see [27]). In this sense, questionnaire ratings reflect that elderly attendees found these type of lectures useful to acquire new information that can have a positive impact in their life, and increased their awareness of how they can improve their brain function and promote brain plasticity through lifestyle modifications.

Recent studies have shown the cognitive and emotional benefits of practicing cognitively stimulating activities in older ages [28,29]. Late-life cognitive activities can influence cognitive reserve and reduce the onset of accelerated memory decline [18]. Specifically, playing a musical instrument is a complex and motivating activity that comprises the coordination of multiple sensory modalities (auditory, visual, and somatosensory) and motor system in a unique way. Previous studies have found that learning to play a musical instrument at an older age can boost executive functions and improve quality of life [30,31]. In this same line, activities such as learning a second language and having a life-long bilingual experience have also proven to be a protective mechanism against age-related cognitive decline [32,33]. As it can be seen in Table 1, some of the lectures included in the series focused on explaining the impact of musical learning or learning a new language on brain function and mood. Interestingly, respondents reported a high to medium interest in learning one of these activities. Furthermore, they also judged with high to medium scores the usefulness and richness of learning these type of activities in older ages.

Furthermore, factors like nutrition, cardiovascular health, physical activity, and sleep quality can influence healthy brain aging. Several studies have shown that the intake of specific nutrients and the practice of physical activity can influence neuronal function and synaptic plasticity by modulating, for example, brain-derived neurotrophic factor [19,20]. In this same line, recent evidence indicates that sleep is not only a fundamental aspect for the replenishment of organisms, but it is also crucial for the appropriate functioning of learning and memory (for reviews see [21,22]). In this study we found that after attending the lectures respondents reported carrying out behavioral changes in these specific domains in order to improve their health. This preliminary data indicates that our strategy, based on offering neuroscientific-based lectures to elderly populations, might be a cost-effective method to promote behavioral changes that lead to healthy brain aging. In this sense, some of the advantages that this type of strategy offers is that it can be delivered to a wide range of public in comparison to other prevention programs which are individualized. Furthermore, as a primary prevention strategy it can also encourage older adults to assume responsibility over their own health and prevent the onset of several diseases which can have very high economic healthcare costs for society [34,35].

Interestingly, some of the reported behavioral changes were positively correlated with the extent to which the respondents modified their perception concerning the learning capacity of older adults. Those who, after attending the lectures, had a more positive perception concerning the ability of older adults to learn new things, also reported more changes in the physical activity and nutritional domains. These results seem to support past studies showing that the sense of control and the perceptions of self-efficacy in the elderly can contribute to successful aging and reduce age related decline [36,37]. This result underscores the importance of promoting self-efficacy and empowering older adults in these types of interventions. Although it was expected that the attendance at a higher number of conferences favored higher changes in attitudes and behaviors towards a healthy lifestyle, a correlational analysis between the number of attended conferences and questionnaire responses did not reveal significant differences. This lack of significance should be interpreted with caution since there was a large variability among the number of attended conferences by the respondents. However, it could be possible that just attending a few conferences is sufficient in order to promote positive attitudes towards healthy aging and behavioral changes. This result seems in accordance with a meta-analysis of preventive strategies in order to maintain independence in older adults which demonstrated that the intensity (number of sessions) of the interventions did not predict better health outcomes [38]. Concerning the age of participants, we just found a significant negative correlation which indicated that older adults with a more advances age reported less interest in learning how to play a musical instrument. Based on this pilot study, we cannot rule out the existence of other significant correlations that we did not detect due to the small sample size.

Based on the results of this pilot study, there are a series of considerations to take into account in future research which aim to further understand the health benefits of these neuroscientific information based-strategies on elderly populations. The results of the present study are based on descriptive information obtained from questionnaire responses. Questionnaire responses are based on a respondent’s subjective experience and a lot of the information reported is based on the recall of certain events. These types of responses are prone to be inaccurate and are not as precise as measuring behaviors directly, so results should be interpreted with caution. Future studies should focus on measuring the impact of these strategies using objective and validated measurements, like daily self-recording of behaviors, the use of standardized health questionnaires or by measuring physiological health indexes before and after attending these types of lectures.

Moreover, future studies should also measure the impact of these types of interventions with a bigger sample size, control for possible inter-individual differences in motivation for change, and calculate response rates. In the current study, we gave the questionnaire to the audience older than 50, however only 32 people sent back the questionnaire to our laboratory, which may bias the responding population towards the most motivated ones. Therefore, caution should be taken in generalizing these results to all attendees to the lectures. In this sense, we were unable to calculate the exact response rate due to the lack of information of the precise number of participants per lecture or their ages. However, lectures were well attended (see above), illustrating the high interest of elderly population for brain-related topics.

It is important for future studies to consider taking a baseline measurement of the attitudes and behaviors targeted by the intervention. Pre-assessment measurements will contribute to control for possible inter-individual differences on baseline levels as well as to measure in a more accurate and precise way the impact of the intervention. Finally, in the current study not all the respondents attended all lectures, so questionnaires were sent back to the laboratory at different times. Due to this limitation we cannot rule out that the time window for behavioral changes to occur was long enough. Further studies should control for this, and more importantly, establish a specific temporal interval (after attending the last lecture) in order to measure whether behavioral changes did actually occur.

## 5. Conclusions

In this pilot study we have found that the promotion of healthy aging through neuroscientific information-based strategies is a promising method to promote healthy brain aging in older adults. The preliminary results of this study indicate that providing information about brain function and plasticity by means of conferences can increase the elderlys’ knowledge about how to maintain and eventually improve brain function through lifestyle modifications. Furthermore, we also found that this type of health promotion strategy potentially encourages positive changes in behaviors related to nutrition, sleep, and realization of physical and cognitive-stimulating activities. It is important to take into account that, according to our findings, behavioral changes are correlated with the elderly’s perception of self-efficacy and empowerment. Future research should study the mid and long term effectiveness of this type of strategies, the persistence of the changes in habits and the impact on the actual health of the elderly community. Longer term programs of conferences are probably a useful strategy to stimulate the persistence of healthy habits.
